# Exosomes: Toward a potential application in bladder cancer diagnosis and treatment

**DOI:** 10.1002/SMMD.20230027

**Published:** 2023-10-30

**Authors:** Xiaowei Wei, Dagan Zhang, Yefei Zhu

**Affiliations:** ^1^ Laboratory Medicine Center The Second Affiliated Hospital of Nanjing Medical University Nanjing China; ^2^ Department of Rheumatology and Immunology Institute of Translational Medicine Nanjing Drum Tower Hospital The Affiliated Hospital of Nanjing University Medical School Nanjing China

**Keywords:** biomarker, bladder cancer, clinical diagnosis, exosome, prognostic monitoring

## Abstract

Bladder cancer (BC) is a prevalent malignant tumor of the urinary system, known for its rapid progression and high likelihood of recurrence. Despite ongoing efforts, clinical diagnosis and treatment of BC remain limited. As such, there is an urgent need to investigate potential mechanisms underlying this disease. Exosomes, which contain a variety of bioactive molecules such as nucleic acids, proteins, and lipids, are regarded as extracellular messengers because they are implicated in facilitating intercellular communication in various diseases and are pivotal in tumor advancement, serving as a promising avenue for such researches. Nevertheless, the heterogeneous nature of BC necessitates further exploration of the potential involvement of exosomes in disease progression. This review comprehensively outlines the biological attributes of exosomes and their critical roles in tumorigenesis, while also discussing their potential applications in regulating the progression of BC involving clinical diagnosis, prognostication and treatment.

## Graphical abstract

Bladder cancer poses a challenge for clinical diagnosis and treatment with the rapid progression and high recurrence rate. Exosomes play a key role in tumor progression. This review comprehensively outlines the biological characteristics of exosomes and their critical roles in tumorigenesis, while also discussing the potential applications in the clinical diagnosis and treatment of bladder cancer.

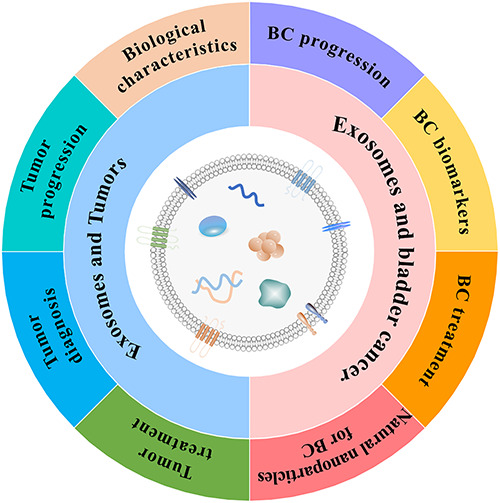

**Key points**

- This paper reviewed the biological characteristics of exosomes and their biological significance in tumor genesis and progression.
- The application prospect of exosomes in bladder cancer (BC) diagnosis and treatment was systematically discussed.
- The potential role of exosomes in the heterogeneity of BC was reviewed.
- A novel idea of using exosomes as the natural nanoparticles in clinical diagnosis and treatment was introduced.

## 1 INTRODUCTION

Bladder cancer (BC) is a prevalent malignancy of the genitourinary system, ranking as the ninth most common malignancy globally. BC can be broadly classified into urothelial BC and non‐urothelial BC based on histopathological criteria. Urothelial carcinoma accounts for nearly 90% of BC cases, with 20% of cases being initially diagnosed as muscle‐invasive bladder carcinoma (MIBC) and carrying a poor prognosis. However, even patients initially diagnosed with non‐muscle‐invasive bladder cancer (NMIBC) may experience disease progression to MIBC, with rates ranging from 10% to 30%.^1^, ^2^ The survival rates for BC have exhibited no significant improvement over the last three decades, as evidenced by approximately 60%–70% of NMIBC patients experiencing disease recurrence post‐surgery.^3^ The long‐term survival rate of BC is intricately linked to the stage of diagnosis. For instance, the 5‐year survival rate of bladder carcinoma in situ is as high as 95.8%, while that of metastatic BC is mere 4.6%. This underscores the criticality of timely and precise diagnosis in determining the prognosis of BC patients.^4^

Currently, the primary diagnostic methods for BC are cystoscopy and cytology.^5^ However, the sensitivity of cytological examination is notably low, particularly for low‐grade tumors, with only a detection rate of 34%.^6^, ^7^ Although cystoscopy is the current gold standard for BC diagnosis, it is an invasive, costly, and poorly tolerated procedure, often resulting in urethral stricture, perforation, bleeding, and infection.^8^ In recent years, exosomes have emerged as a prominent tool in the field of oncology. Heterogeneous cells secrete membrane‐bound vesicles that contain abundant nucleic acid, protein, and lipid contents.^9^ These exosomes, derived from tumor cells, act as mediators of bioactive molecules and play a role in inducing the tumor microenvironment (TME), including the formation of pre‐metastasis niches.^10^, ^11^ Furthermore, exosomes offer a potential source of novel biomarkers for liquid tumor biopsy and may provide a safe and targeted alternative approach for tumor therapy due to their low immunogenicity and circulatory advantage.^12^

Despite the development of exosome‐based mechanisms and applications in various diseases, their utilization in BC remains in nascent stages. This review endeavors to elucidate the biological significance of exosomes in tumor progression and their prospective clinical applications in the diagnosis and treatment of BC.

## 2 EXOSOMES AND TUMOR

### 2.1 Biological characteristics of exosomes

**FIGURE 1:**
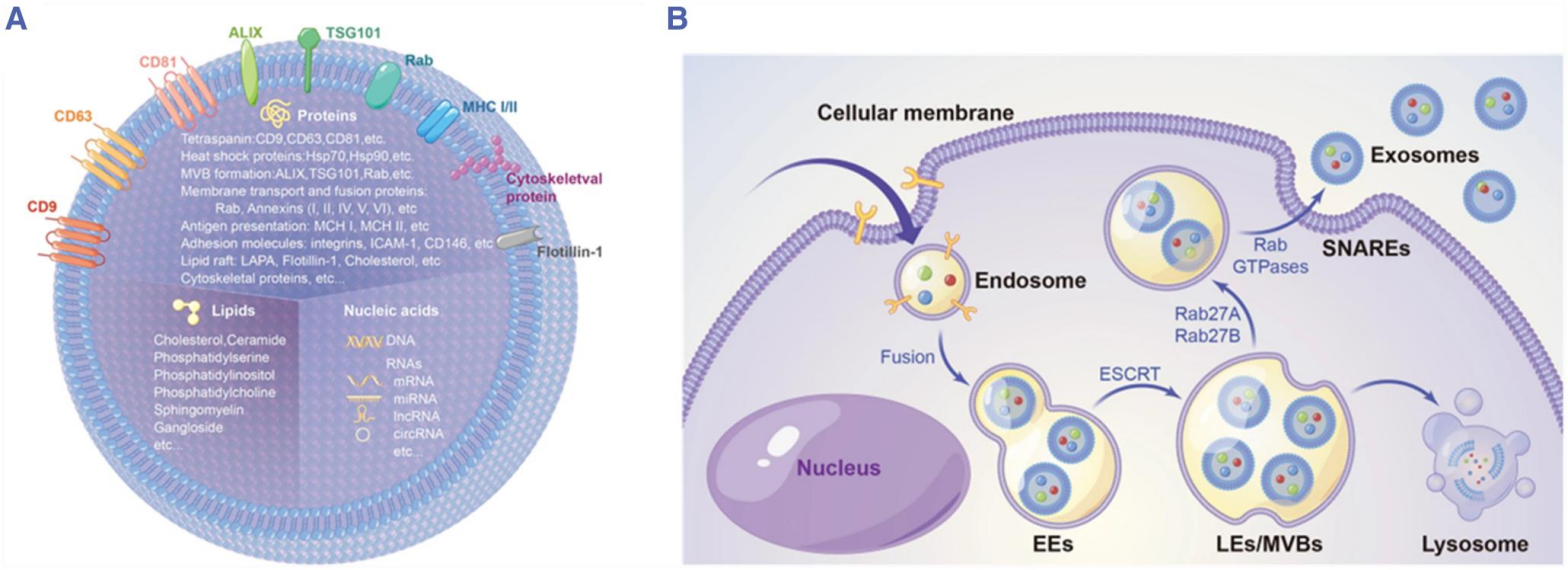
(A) Characteristics and contents of exosome; (B) biogenesis and secretion of exosomes. Reproduced under terms of the CC‐BY license.^16^ Copyright 2021, The Authors, published by Springer Nature.

Exosomes are extracellular vesicles (EVs) with diameter of 30–150 nm, which can mediate biological activity. They are composed of bilayer lipid coating and rich in biomolecules including nucleic acid, proteins, and lipids, which can reflect the characteristics of parent cells.^11^, ^13^ Due to the lipid bilayer, exosomes are stable, resistant to the degradation of circulating RNases or other enzymes, and can maintain the integrity of their contents for a longer time. The biogenesis and release of exosomes are a complex multi‐step process. Early endosomes are formed by endocytosis of the plasma membrane and further mature as multivesicular body (MVB). During this process, exosomes are secreted in the form of intracavitary vesicles after fusion of the MVB membrane with the plasma membrane and serve as the key mediators of intercellular communication^14^, ^15^, ^16^ (Figure 1).

**FIGURE 2:**
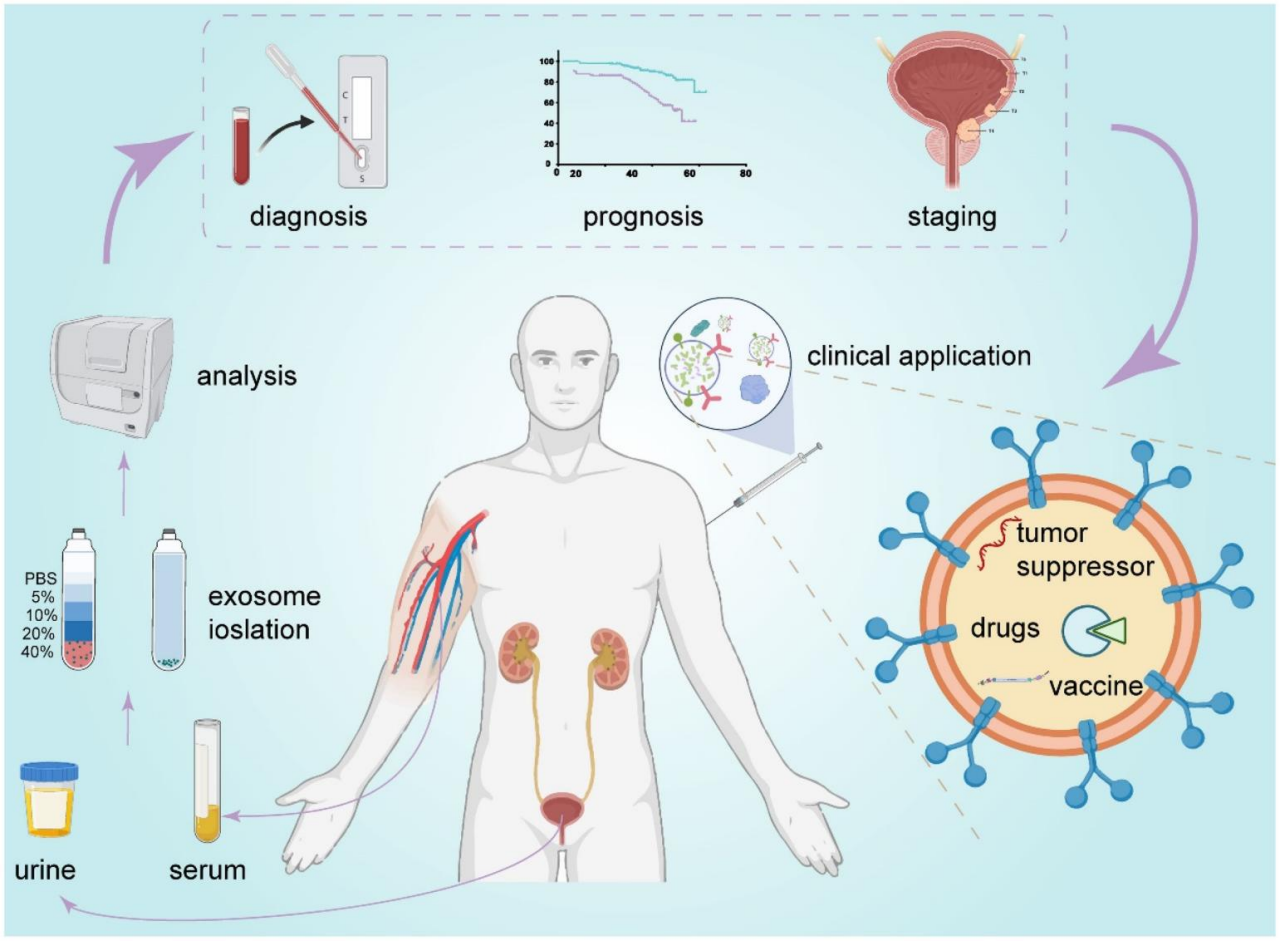
The clinical application prospect of exosomes in bladder cancer. Reproduced under terms of the CC‐BY license.^23^ Copyright 2021, The Authors, published by Frontiers Media S. A.

More and more studies have shown that exosomes are involved in many important physiological processes, including neutrophil chemotaxis, extracellular matrix remodeling, immunity, inflammation, and central nervous system physiology, etc.^17^ Besides, it's reported that exosomes are related to a wide range of disease pathology, including different types of kidney disease, Parkinson's disease, Alzheimer's disease, and tumor, etc.^18^ During tumorigenesis, exosomes play a key role in tumor progression by increasing the invasiveness and migration, promoting angiogenesis, activating cancer‐related fibroblasts, and enhancing cell proliferation, thus participating in tumor formation, metastasis, immune escape and drug resistance, etc.^19^, ^20^, ^21^, ^22^, ^23^ (Figure 2).

### 2.2 Exosomes and tumor progression

TME is a key element of tumor generation. The interaction between cells and the microenvironment can promote the occurrence and progress of tumors.^24^ Studies have shown that exosome‐derived bioactive molecules secreted by tumor cells can generate the pre‐metastasis niche by reshaping the TME. This complex process involves the binding of tumor‐cell‐derived exosomes to the target organ stromal cells, leading to cell reprogramming, activation of signaling pathways, and ultimately the establishment of a pre‐metastatic microenvironment in the target organ, providing a prerequisite for promoting tumor growth and metastasis.^25^, ^26^ Among them, tumor‐cell‐derived exosomes are considered as the crucial mediators of cell‐cell interactions in the TME, which are involved in variety of tumorigenic mechanisms.

#### 2.2.1 Angiogenesis promotion

Tumor occurrence and progression is a dynamic, multi‐step process that requires adequate blood conduction to provide the continuous nutrients and oxygen to maintain the rapid growth and reproduction of tumor cells. Tumor cells promote angiogenesis by activating endothelial cells.^27^ Exosome‐associated mRNA encodes vascular endothelial growth factor, fibroblast growth factor, angiopoietin‐1, ephrin A3, matrix metallopeptidase 2, matrix metallopeptidase 9 and azurocidin 1, etc. While microRNAs (miRNAs) are involved in regulating the transcription and metabolic processes, promoting the generation of tumor vascular networks.^28^, ^29^, ^30^, ^31^ This is not only a necessary condition for the normal growth of tumor tissue, but also an important pathway for tumor invasion.^32^

#### 2.2.2 Epithelial‐mesenchymal transition

**FIGURE 3:**
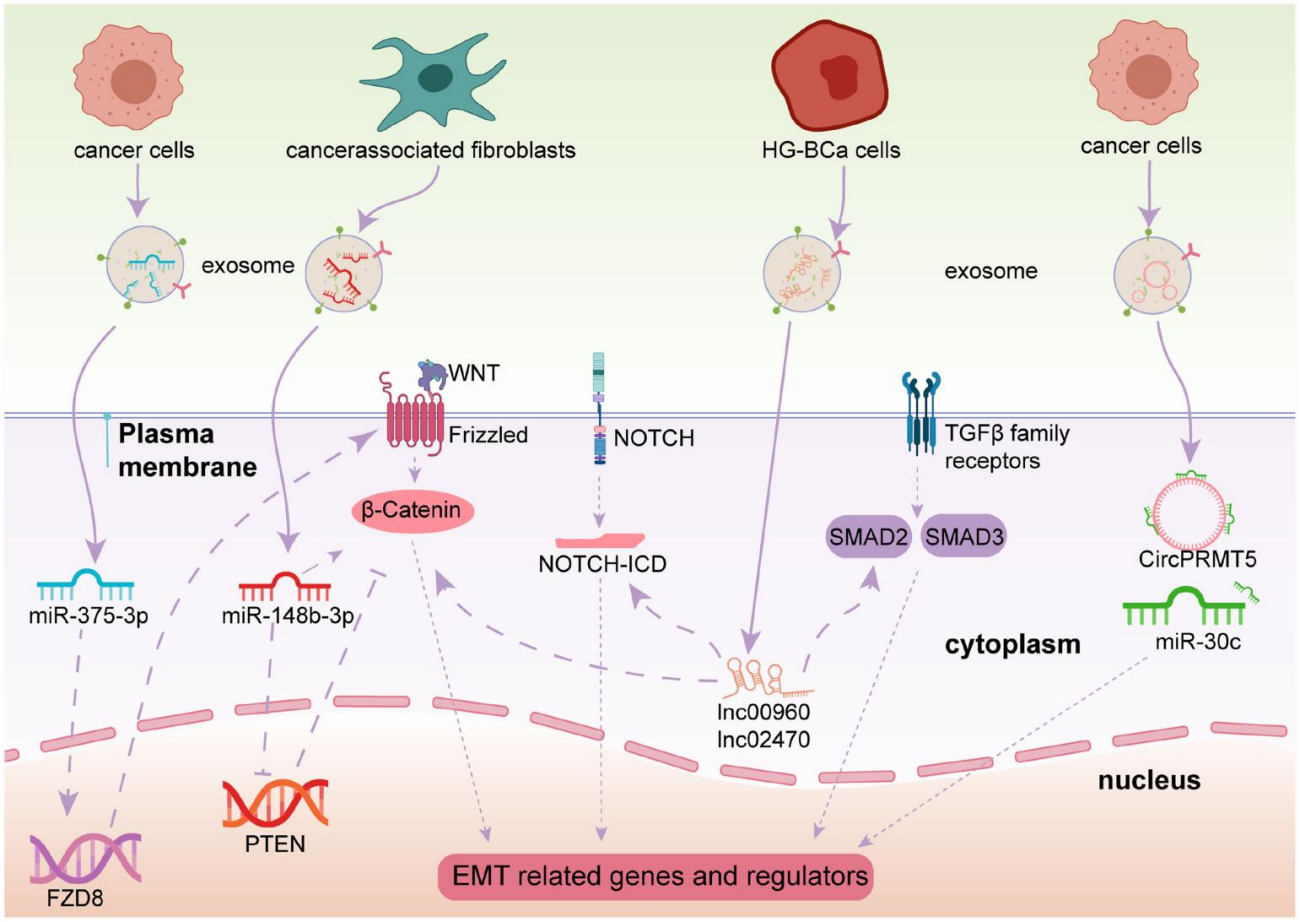
The role of exosome‐derived non‐coding RNAs in EMT processes. Reproduced under terms of the CC‐BY license.^23^ Copyright 2021, The Authors, published by Frontiers Media S. A. EMT epithelial‐mesenchymal transition.

It is the transformation of epithelial cells into mesenchymal cells, which involves in embryogenesis, wound healing and malignant progression of tumors. Intercellular and extracellular matrix interactions are reshaped, resulting in reduced adhesion between epithelial cells and the basement membrane, further activating the new transcriptional processes that facilitate their mesenchymal transformation. In tumor progression, epithelial‐mesenchymal transition (EMT) promotes tumor formation and metastasis while increasing the tolerance to clinical intervention. Exosomes promote tumor invasion and migration by regulating extracellular matrix and EMT process (Figure 3). Tumor exosomes acting on the urothelial cells have been found to induce EMT.^33^, ^34^

#### 2.2.3 Premetastatic niches formation

This is a key molecular event for tumor target organ/tissue metastasis.^35^ Primary tumors release biomolecules that migrate to preferred metastatic targets and dynamically reshape these sites before metastasizing to distant organs. Premetastatic niches are defined as the molecular and cellular changes in specific organs/tissues. Distant metastasis of tumors is facilitated by the colonization of target organs/tissues by circulating tumor cells,^36^ which involves the release of initial tumor‐derived exosomes into the circulatory system and subsequent escape from the vascular bed to the distant secondary organs.^27^, ^37^ In this process, the organ affinity exosomes exhibit is key to their targeting of organ tissues. By modifying the function of target cells through content biomolecules, exosomes play an important role in tumor invasion and metastasis.

#### 2.2.4 TME regulation

An increasing number of studies have demonstrated that tumor progression is the result of autocrine and paracrine communication between tumor cells in the microenvironment, which tend to secrete large amounts of exosomes.^38^, ^39^ For example, cancer‐associated fibroblasts (CAFs) induced by exosome secretion of miR‐9 and telomerase reverse transcriptase (hTERT) support tumor cell proliferation and increase their drug resistance. Signaling pathways activation is also one of the pathways that exosomes participate in the regulation of TME. For example, transforming growth factor‐β (TGF‐β)1 promotes the differentiation of human umbilical cord mesenchymal stem cells into CAFs and activates the TGF‐β/Smad pathway.^40^ In the communication between normal stromal cells and tumor cells, exosomes may stimulate another signaling pathway, such as nuclear factor κB and epidermal growth factor receptor signaling pathway, which also play a critical role in tumor proliferation and migration. After exposure to tumor exosomes, endothelial cells showed enhanced proliferation, migration, and tubular formation. In addition, tumor cell exosomes have been proved to promote cancer progression by reducing the immune response. Notably, some specific exosomes may even reduce the cytotoxicity of natural killer cells and T cells. Tumor‐derived exosomes can also influence autologous cells through autocrine, stimulating tumor cell invasion and migration while reducing the adhesion by enhancing MMP 9 or chemokine receptor 4.^41^

### 2.3 Exosomes and tumor diagnosis

Exosomes are secreted by various types of parent cells and are produced by endosomal budding. The lipid bilayer membranes protect bioactive molecules from enzymatic degradation in the extracellular environment through membrane fusion and internalization. Bioactive molecules, such as nucleic acids (non‐coding RNA, messenger RNA, etc.) and proteins, are selectively classified, packaged and delivered to cells to participate in immune response, antigen presentation, and intercellular communication, and have become novel mediators for tumor progression in the past decade.^42^ The abundance of exosomes in blood and urine makes them a potential source of effective biomarkers for tumor diagnosis and detection.^43^

In recent years, exosome‐derived biomarkers as potential candidates for primary disease diagnosis and prognostic monitoring have attracted extensive attention in laboratory diagnosis. As an important liquid biopsy technique, the detection of blood‐derived and urinal‐derived exosomes has an ideal clinical application prospect in the in vitro diagnosis of tumors due to the unique advantages of non‐invasive and simple operation, as well as the comprehensive display of complete genetic information of tumor patients. Compared to the traditional tissue biopsies, liquid biopsies allow easy and quick specimen access with less patient trauma. In the diagnosis and monitoring of tumors, liquid biopsy makes it easier to ensure the standardization and accuracy of the procedures, so that the analysis results have a better repeatability. Exosomes present different levels of enrichment in blood and urine based on the different tumor characteristics. Therefore, selective sampling can be used to detect the target exosome‐derived biomarkers to improve the clinical diagnostic efficacy. Urinary exosome‐related biomarkers have better applications in the diagnosis and monitoring of urinary tumors, while blood‐derived exosome markers may be a better choice for the non‐urinary tumors. Exosome‐related biomarkers show promising clinical application prospects in improving the diagnostic sensitivity and accuracy, especially for the low‐grade tumors.

### 2.4 Exosomes and tumor treatment

In recent years, many studies have reported the applications of exosomes in tumor therapy. By exploring the effective biological effects and the reduction of toxicity of traditional drugs, exosomes can provide an important laboratory foundation for tumor research.

#### 2.4.1 Exosomes and tumor immunity

Tumor cells and their TME‐derived exosomes directly or indirectly influence the tumor progression through cellular action and TME regulation, respectively. Therefore, blocking the production of exosomes and the mediated tumor‐TME cellular signaling pathway, and eliminating the exosome‐specific active contents, have been proposed as the therapeutic strategies to inhibit the tumor progression. In addition, the efficacy of exosomes in immunotherapy may hold promise for tumor treatment. Recent studies have shown that immunotherapy, especially the emergence of immune checkpoint blocking (i.e., PD‐1/PD‐L1), has become a new breakthrough in cancer treatment. Tumor‐derived exosomes deliver functional PD‐L1 and inhibit the immune response.^44^ Therefore, systemic anti‐tumor immunity and memory can be induced by the inhibition of exosome PD‐L1.^45^ To date, studies based on exosomes promoting anti‐tumor immunity have begun to explore their safety and efficacy in humans.

#### 2.4.2 Exosomes as tumor vaccines

Tumor‐derived exosomes follow the antigen presentation pathway and can be used as cell‐free vaccines.^46^ As key regulators in biological systems, non‐coding RNAs influence the differentiation, maturation and function of immune cells.^47^ For example, non‐coding RNA‐modified exosomes target interleukin‐6 (IL‐6), interleukin‐17 (IL‐17), interleukin‐1B (IL‐1B), TGF‐β, interferon‐γ, and Toll‐like receptor 4, inducing dendritic cells to mature and enhance their immunostimulatory capacity.^48^ Therefore, tumor‐derived exosomes modified with specific non‐coding RNA are expected to play an important role in the treatment of BC.

#### 2.4.3 Exosomes as drug delivery vectors

Studies have shown that therapeutic nanoparticles can be loaded into liposomes to increase the drug concentration in target tissues while reducing the toxic effects on normal tissues.^49^, ^50^ Numerous studies have extensively endeavored to ascertain the most suitable exosome donor cell type, while also focusing on the efficient exosome isolation, engineered exosome production, and storage to facilitate the abundant accumulation of exosomes from non‐specific locations toward the targeted tissues. Further major challenges include unsatisfactory preservation and delivery efficiency of exosome structural integrity during drug loading. Clarification of these issues will enhance the application prospect of exosomes as drug delivery vectors.

## 3 EXOSOME AND BC

### 3.1 Exosomes in BC progression

**FIGURE 4:**
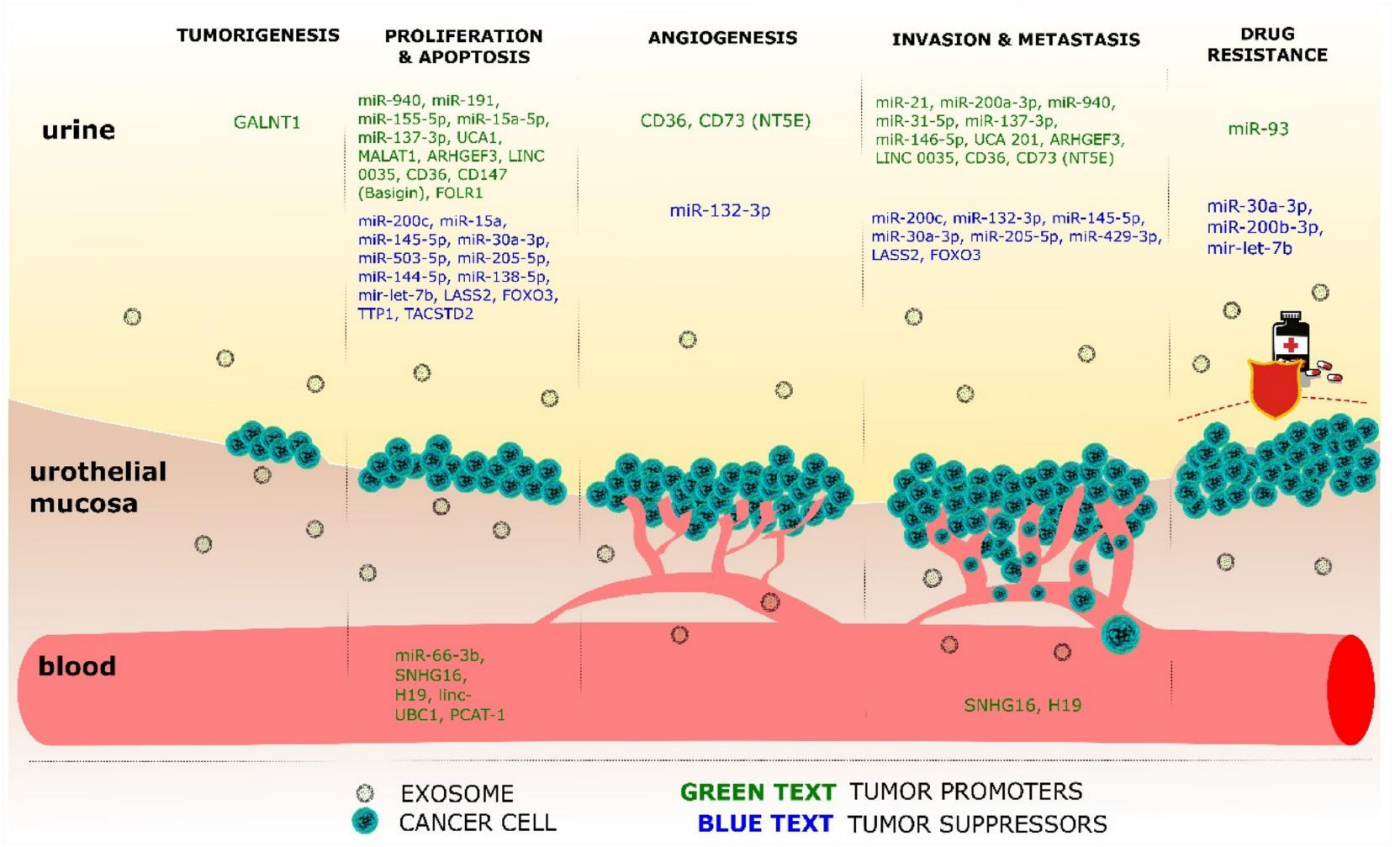
Role of urinary and blood exosome‐derived biomarkers in tumorigenesis at different stages of bladder cancer. Reproduced under terms of the CC‐BY license.^80^ Copyright 2021, The Authors, published by MDPI.

A number of studies have demonstrated that BC‐derived exosomes are similar to exosomes from other tumors, participating in the regulation of biological processes such as cell proliferation, migration, invasion, apoptosis and angiogenesis (Table 1),^51^, ^52^, ^53^, ^54^, ^55^, ^56^, ^57^, ^58^, ^59^, ^60^, ^61^, ^62^, ^63^, ^64^, ^65^, ^66^, ^67^, ^68^, ^69^, ^70^, ^71^, ^72^, ^73^, ^74^, ^75^, ^76^, ^77^, ^78^, ^79^ playing an important role in the tumorigenesis and progression at different stages of BC^80^ (Figure 4). Yang et al. showed that BC exosomes induced the proliferation of moderately differentiated 5637 BC cells and poorly differentiated T24 bladder transitional cells in a dose and time dependent manner. They found that after the BC exosomes treatment, the expressions of anti‐apoptotic genes B‐cell lymphoma/leukemia‐2 (BCL‐2) and Cyclin D1 were increased, while pro‐apoptotic genes Bax and Caspase3 decreased.^81^ Xu et al. reported that exosomes down‐regulate the expression of BCL‐2 and MCL‐1 by transporting miR‐29c in BC, inducing BIU‐87 cell apoptosis.^82^ Beckham et al. found that exosomes isolated from the urine of patients with high‐grade radical BC could promote the migration and invasion of 5637 cells cultured in vitro and stimulate the angiogenesis of human umbilical vein endothelial cells.^83^ Franzen et al. showed that BC exosomes induced EMT in urothelial cells. Compared with controls, urothelial cells exposed to the invasive BC cell lines or bladder patient‐derived exosomes showed the decreased expression of epithelial markers and increased expression of mesenchymal markers.^33^ This could explain the interaction between urinary exosomes and bladder urothelial cells, as well as the fact that the former induces the latter to undergo transformation, making them at a higher tumorigenic risk. Ostenfeld et al. demonstrated that silencing Rab27a and Rab27b members of the Rab family could down‐regulate the expression of miR‐23b and miR‐921 in BC cells to reduce cell invasion, indicating that exosome‐mediated tumor‐suppressor miRNA can be coordinated to inhibit the progression of tumor metastasis.^82^

**TABLE 1:** Potential role and mechanism of BC‐derived exosomes.

Type of marker	Source	Markers	Mechanism/biological function	References
miRNA	Urine	miR‐let‐7b	Promotes apoptosis; suppresses drug resistance	51, 52
		miR‐29b	Suppresses tumor growth and metastasis	51, 52
		miR‐200c	Suppresses EMT, proliferation and invasion	51, 52, 53
		miR‐21	Promotes EMT; enhances cell motility and proliferation; suppresses apoptosis	51
		miR‐31‐5p	Promotes migration and invasion	53
		miR‐940	Promotes proliferation, migration and invasion	52
		miR‐93	Promotes drug resistance; suppresses cisplatin‐induced apoptosis	52
		miR‐205‐5p	Promotes apoptosis; suppresses EMT and invasion	52, 54
		miR‐132‐3p	Promotes angiogenesis, migration; suppresses invasion and EMT	53
		miR‐30a‐3p	Suppresses autophagy; promotes chemosensitivity to cisplatin; suppresses invasion	52, 55
		miR‐138‐5p	Promotes apoptosis	55
		miR‐145‐5p	Suppresses proliferation and migration	56
		miR‐15a‐5p, miR‐66‐3b	Promotes proliferation	53, 57
		miR‐155‐5p, miR‐191	Suppresses apoptosis	52, 53
		miR‐200a‐3p, miR‐146‐5p	Promotes invasion	52, 55
		miR‐15a, miR‐503‐5p, miR‐144‐5p	Suppresses proliferation	52
	Blood	miR‐23b	Suppresses EMT induces G0/G1 cell cycle arrest and apoptosis;	56
		miR‐133b	Promotes apoptosis; suppresses Bcl‐w and Akt1 protein expression	58
		miR‐375‐3p	Suppresses proliferation and invasion	59
		miR‐29c	Suppresses proliferation	60
lncRNA	Urine	MALAT1	Suppresses apoptosis	61, 62
		HOTAIR, UCA 201, LINC 0035	Promotes migration and invasion	61, 63
	Blood	LINC‐UBC1, PCAT‐1	Promotes proliferation	62, 64
		SNHG16	Promotes proliferation, migration and invasion; activates the Wnt/beta‐catenin pathway	64
		H19	Promotes proliferation, EMT and metastasis	65
	Cells	UCA1	Promotes tumor growth and progression	61, 66
		LNMAT2, BCYRN1	Promotes HLEC tube formation and migration; enhances tumor lymphangiogenesis	67, 68
		LINC00960, LINC02470	Enhance the cell viability, migration, invasion and clonogenicity	69
mRNA	Urine	GALNT1	Maintenance of bladder cancer stem cells and bladder tumorigenesis	70, 71
		LASS2	Suppresses cancer cell invasion and proliferation	70, 71
Protein	Urine	Alpha 1‐antitrypsin	Suppresses apoptosis; immunity regulation	72
		MAGEB4	Promotes tumorigenesis and proliferation	73
		NMP22	Promotes proliferation; part of nuclear mitotic apparatus	73
		FOLR1	Promotes proliferation; promotesnucleic acid synthesis	74
		TACSTD2, TTP1	Promotes apoptosis	74, 75, 76, 77
		H2B1K	Suppresses tumorigenesis; regulates response to DNA damage	72
	Cells	H3F3A, H3F3B	Replaces conventional H3 in a wide range of nucleosomes in active genes	76, 78
		LAMB3, LAMC2	Mediates the attachment and migration of cells	76, 78
		PYGL	Important allosteric enzyme in carbohydrate metabolism	76, 78
	Cells/Urine	CD147 (Basigin)	Promotes proliferation	79
		5T4	Promotes EMT and migration	79
		CD36, CD44	Promotes proliferation, migration and angiogenesis	79
		CD73 (NT5E)	Promotes angiogenesis, metastases and invasion	79

Abbreviations: EMT, epithelial‐mesenchymal transition; HLEC, human lymphatic endothelial cell.

### 3.2 Exosomes as BC biomarkers

Exosomes are involved in the transformation of a variety of diseases, including the occurrence, progression and clinical staging of tumors, providing a deeper understanding of the pathogenesis of BC.^84^ Many research groups have explored the differential expression of non‐coding RNA, mRNA and protein in exosomes isolated from BC cell lines or urine, providing a research basis for the discovery of BC exosomal biomarkers and the establishment of tumor library (Table 2).^19^, ^52^, ^55^, ^57^, ^63^, ^66^, ^76^, ^83^, ^85^, ^86^, ^87^, ^88^, ^89^, ^90^, ^91^, ^92^, ^93^, ^94^, ^95^, ^96^, ^97^, ^98^, ^99^, ^100^, ^101^, ^102^, ^103^ Although the search for exosomal biomarkers for BC is still in its infancy, some studies have showed the exciting future prospects.

**TABLE 2:** Potential of exosomal biomarkers as diagnostic and prognostic tool in BC.

Type of marker	Source	Markers	Biomarker potential	References
miRNA	Urine	miR‐940	Prognosis	85
		miR‐141‐3p, miR‐200a‐3p, miR‐205‐5p	Prognosis	86
		miR‐375, miR‐146a	Diagnosis, prognosis	57
		miR‐214	Prediction of NMIBC recurrence	87
		miR‐22‐3p, miR‐200a‐3p	Prediction of NMIBC recurrence	19
		miR16, miR200c, miR205, miR21, miR221, miR34a	Prediction of NMIBC recurrence	88
	Blood	miR‐152	Prediction of NMIBC recurrence	89
		miR‐422a‐3p, miR‐486‐3p, miR‐103a‐3p, miR‐27a‐3p	Prediction of MIBC survival	90
	Cells	miR‐21‐5p, miR‐let‐7i‐3p	Prediction of response to chemotherapy	91
lncRNA8	Urine	UCA1‐201, HOTAIR, HYMA1, MALAT1	Diagnosis	92
		HYMA1, LINC00477, LOC100506688, OTX2‐AS1	Prognostic	63
		HOTAIR, HOX‐AS2	Diagnosis, prognosis	93
		MALAT1, PCAT1, SPRY4‐IT1	Diagnosis, prognosis	94
	Blood	UCA1	Diagnosis	66
		PTENP1	Diagnosis	95
		PCAT‐1, UBC1, SNHG16	Diagnosis, prognosis	55
		H19	Diagnosis, prognosis	96
mRNA	Urine	LASS2, GALNT1	Diagnosis	52, 97
		SOX2, OCT4	Diagnosis, prognosis	93
Protein	Urine	Apo A1, CD5L, FGA, FGB, FGG, HPR, HP	Diagnosis	76
		HEXB, S100A4, SND1	Diagnosis	98
		Resistin, GTPase NRas, MUC4, EPS8L1, EPS8L2, EHD4, G3BP, RAI3, GSA	Diagnosis	83, 99
		Periostin	Prognosis	100
		Alpha‐1 antitrypsin, histone H2B1K	Diagnosis, prognosis	76
		ApoB	Diagnosis, prognosis	57
	Cells	Vimentin, CK2, HDGF, annexin 2, moesin	Prognosis	101
	Cells/Urine	EDIL‐3	Diagnosis	102
		β1 and α6 integrins, CD36, CD44, CD73, CD10, MUC1, basigin, 5T4	Diagnosis	103

Abbreviations: BC, bladder cancer; MIBC, muscle‐invasive bladder carcinoma; NMIBC, non‐muscle‐invasive bladder cancer.

#### 3.2.1 Non‐coding RNAs

**FIGURE 5:**
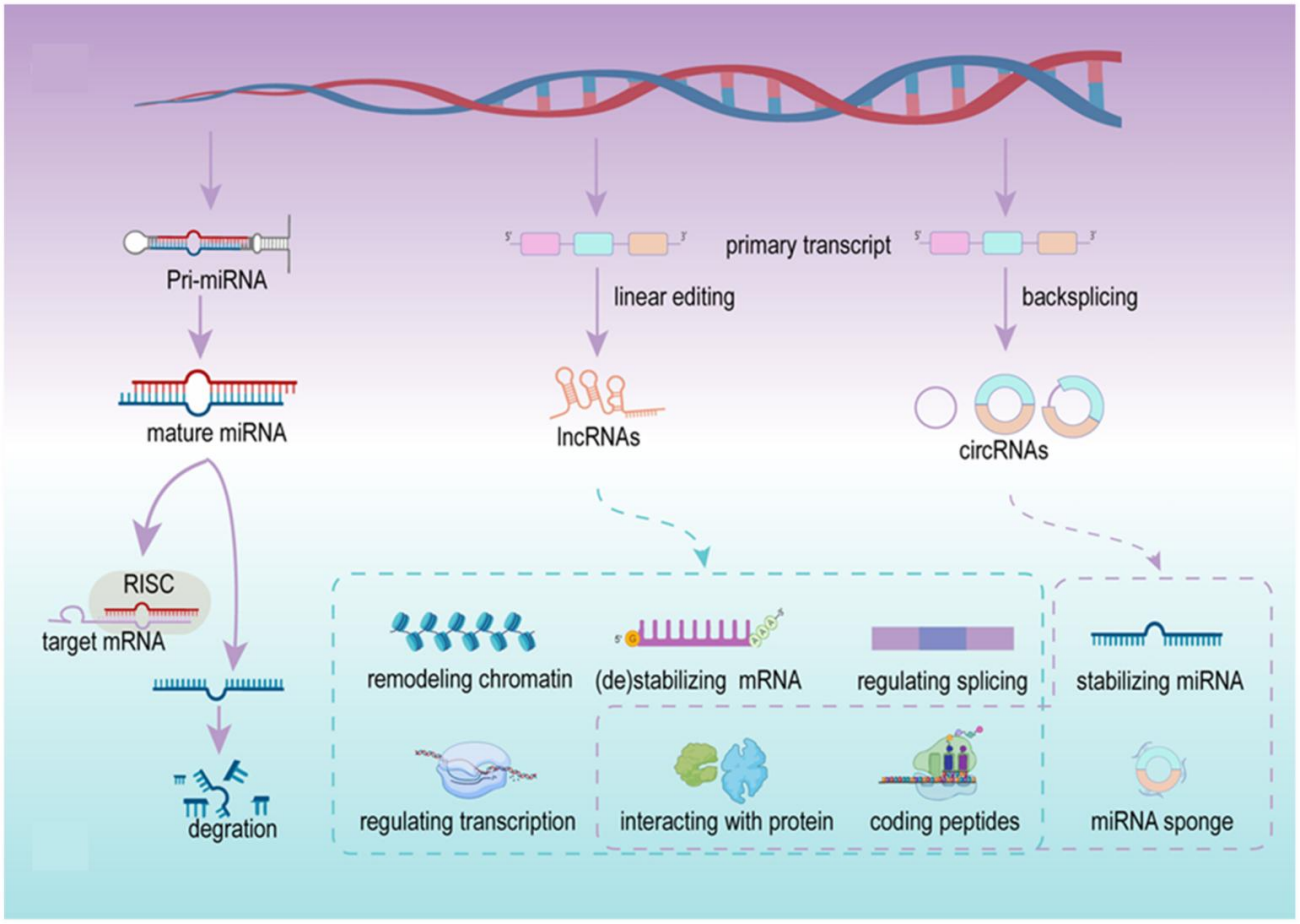
Biogenesis and potential roles of exosome‐derived noncoding RNAs in bladder cancer. Reproduced under terms of the CC‐BY license.^23^ Copyright 2021, The Authors, published by Frontiers Media S. A.

**FIGURE 6:**
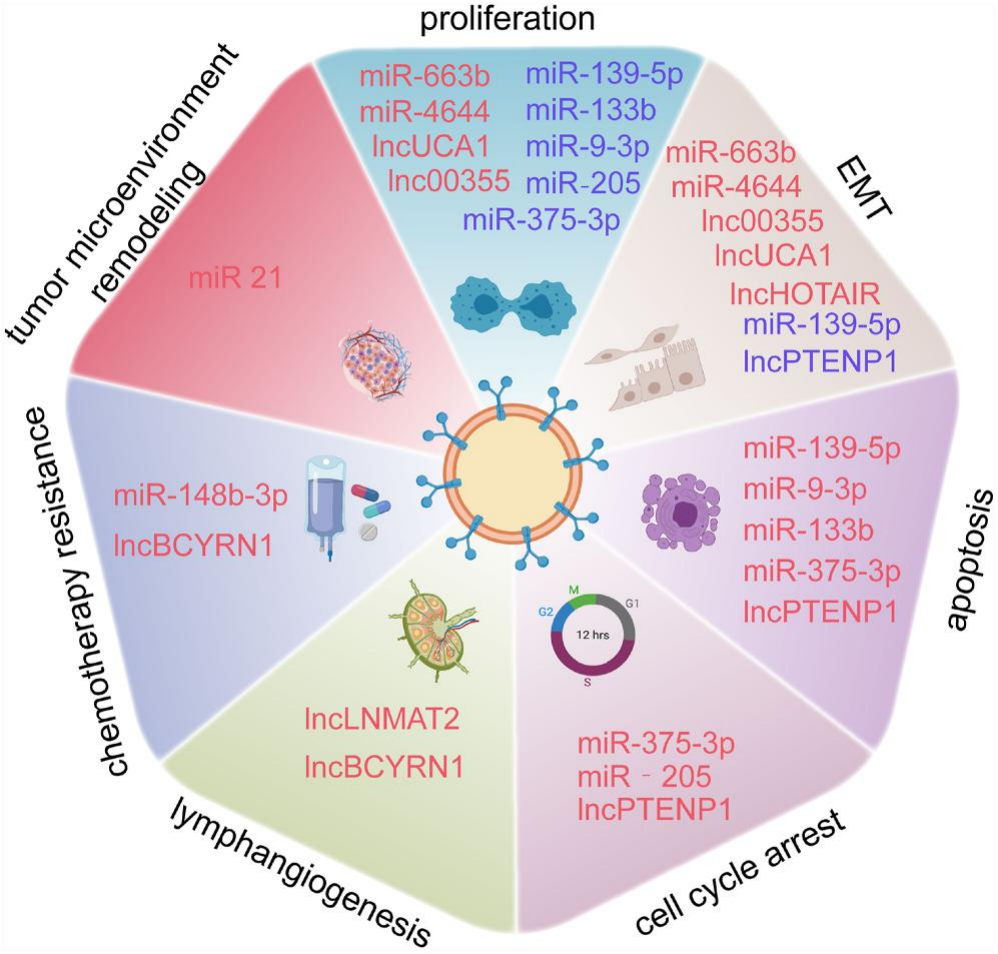
Biological properties of non‐coding RNAs. Reproduced under terms of the CC‐BY license.^23^ Copyright 2021, The Authors, published by Frontiers Media S. A.

Exosomes are found to be rich in content molecules, among which a large number of different types of non‐coding RNAs are involved in the regulation of cell activity and intercellular communication.^104^ The types and amounts of non‐coding RNAs are influenced by the surrounding microenvironment and parent cells, and are delivered and utilized as the recipient cells (Figure 5). Current reviews on non‐coding RNAs from BC‐derived exosomes mainly focus on miRNAs, long‐chain non‐coding RNAs and circular RNAs, which are involved in the regulation of tumor cell proliferation, invasion, apoptosis, cell cycle arrest, angiogenesis and lymphangion genesis. Due to the protective bilayer membrane, bioactive substances in exosomes achieve the resistance to degradation and stability, which are important advantages of exosomes as biomarkers^105^ (Figure 6). By reviewing the previous studies, the relationship between exosomal non‐coding RNAs and BC can be more clearly identified. Therefore, exosome derived non‐coding RNAs are expected to be the potential diagnostic biomarkers and therapeutic targets. Moreover, exosomal non‐coding RNAs can also be used to predict the patient survival parameters such as cancer‐specific mortality, relapse‐free survival, overall survival, and disease‐free survival. Tong et al. collected information from studies reporting survival data and evaluated 9 exosome non‐coding RNAs that were identified as the prognostic biomarkers.^23^

#### 3.2.2 Messenger RNAs

Perez et al. analyzed exosomal RNAs from BC patients and controls using a whole‐transcriptome array and found that there was 55% overlap between BC patients and controls among 4102 transcripts. Based on the differential expression of patient and the control samples, 15 genes were selected for further PCR analysis at eight sites. They found that two genes, GALNT1 and LASS2, were only present in BC patients, while two genes, ARGHGEF39 and FOXO3 only present in the control samples.^52^

#### 3.2.3 Protein biomarkers

Exosome membranes are rich in transmembrane proteins and cytoplasmic proteins, some of which are considered as universal exosome markers regardless of cell origin, such as CD9, CD63, CD81, members of the quaternary transmembrane protein family, and endosomal sorting complex required for transport complex associated with biogenesis. Welton et al. conducted proteomic studies on exosomes of HT1376 bladder carcinoma cells by mass spectrometry and selected 18 proteins for western blot and flow cytometry verification. Flow cytometry analysis of protein expression in three patients with BC and four healthy controls showed that the increased expression in BC included MUC1, integrin β1, integrin α6, CD36, CD44, CD10, 5T4, basigin, and CD73.^76^ Smalley et al. identified 307 proteins in urinary exosomes of BC patients and control patients by mass spectrometry, among which 8 proteins showed increased expression of 38 proteins in BC exosomes, while 5 proteins associated with the epidermal growth factor receptor pathway, the alpha subunit of GsGTP binding protein, Resistin, and retinoic acid‐induced protein.^99^ Chen et al. evaluated urinary exosomal protein expression in BC patients and inguinal hernia using mass spectrometry. They analyzed 2964 urinary exosomal proteins, of which 168 proteins were differentially expressed in patients with BC and inguinal hernia, and 17 proteins were also upregulated in the patients with BC in Welton et al. Furthermore, this work identified 7 proteins (Apo A1, CD5L, FGA, FGB, FGG, HPR, and HP) that were differentially enriched in low and high grades of BC.^73^ Jeppesen et al. found several differentially expressed proteins associated with metastasis potential in BC exosomes, and the increase of some proteins in metastatic BC was associated with EMTs, including vimentin, CK2α, HDGF, annexin 2, moesin, etc.^101^

### 3.3 Exosomes and BC treatment

**FIGURE 7:**
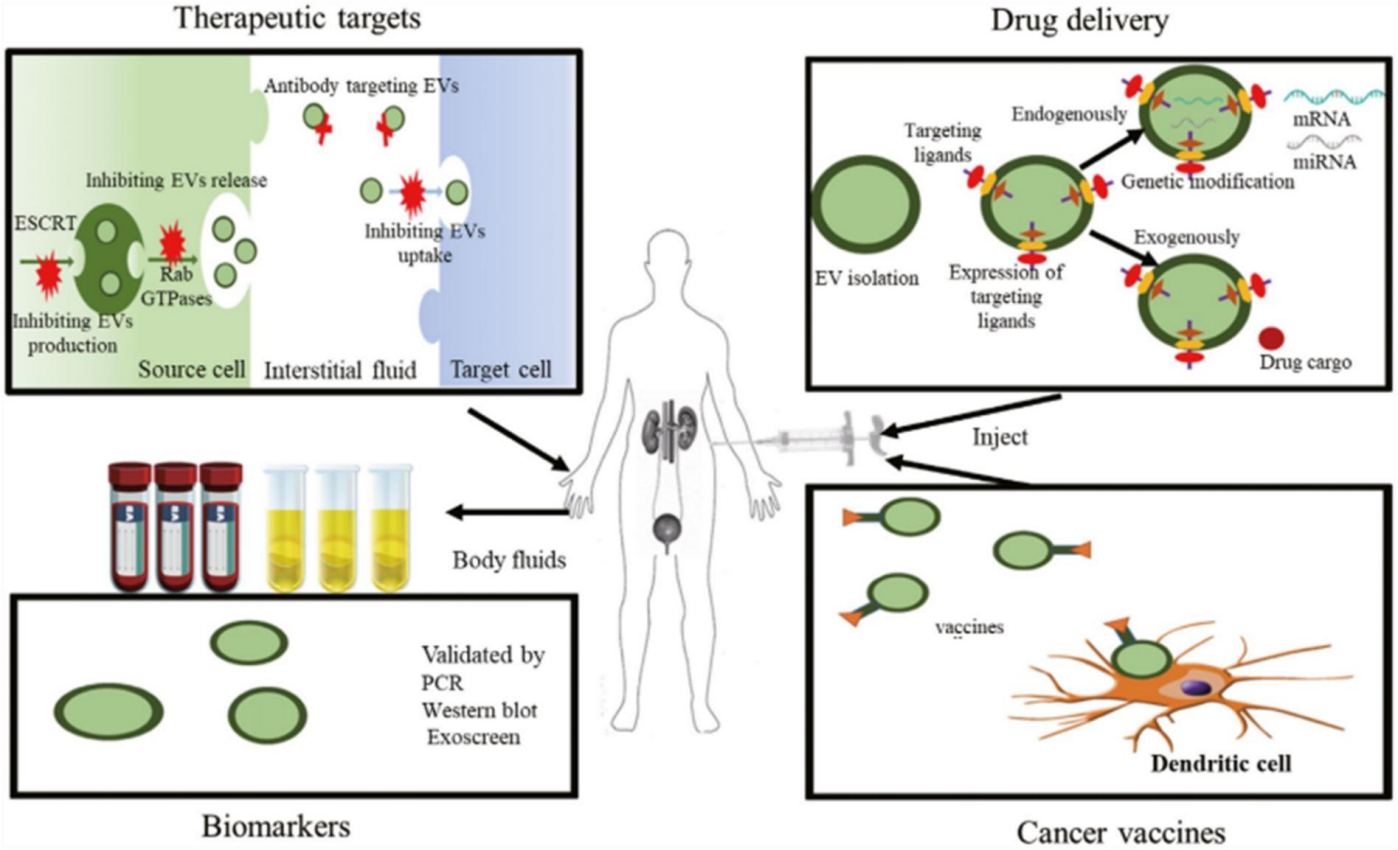
Application prospect of exosomes in urological cancer. Reproduced under terms of the CC‐BY license.^107^ Copyright 2019, The Authors, published by John Wiley and Sons.

Due to the small size and histocompatibility of exosomes, the biomolecules inside the exosomes are enveloped by bilayer lipid membranes to avoid degradation by RNases in the circulation.^106^ Therefore, they can be used as delivery vectors targeting tumor cells^107^ (Figure 7). Franzen et al. demonstrated that BC cells interacted with exosomes and internalized through receptor‐mediated endocytosis.^108^ There is precedent for untargeted delivery of drugs through the bladder. However, the adverse effect of untargeted drug delivery on normal epithelial cells surrounding tumor cells is a possible concern. It was found that BC cells absorbed exosomes more readily (≥50 times) than normal urothelial cells.^109^ Polo‐like kinase 1 siRNA was electro‐perforated into human embryonic kidney cell‐derived exosomes and delivered to BC cells in vitro. The expression of PLK1 mRNA in BC cells was significantly reduced after treatment with the PLK1 siRNA exosomes. This work supported the idea that exosomes could deliver siRNA to the target cells. However, the current research progress in this area is still limited, and more exploration is needed to advance the treatment of exosomes in BC.

### 3.4 Exosomes as natural nanoparticles in BC

**FIGURE 8:**
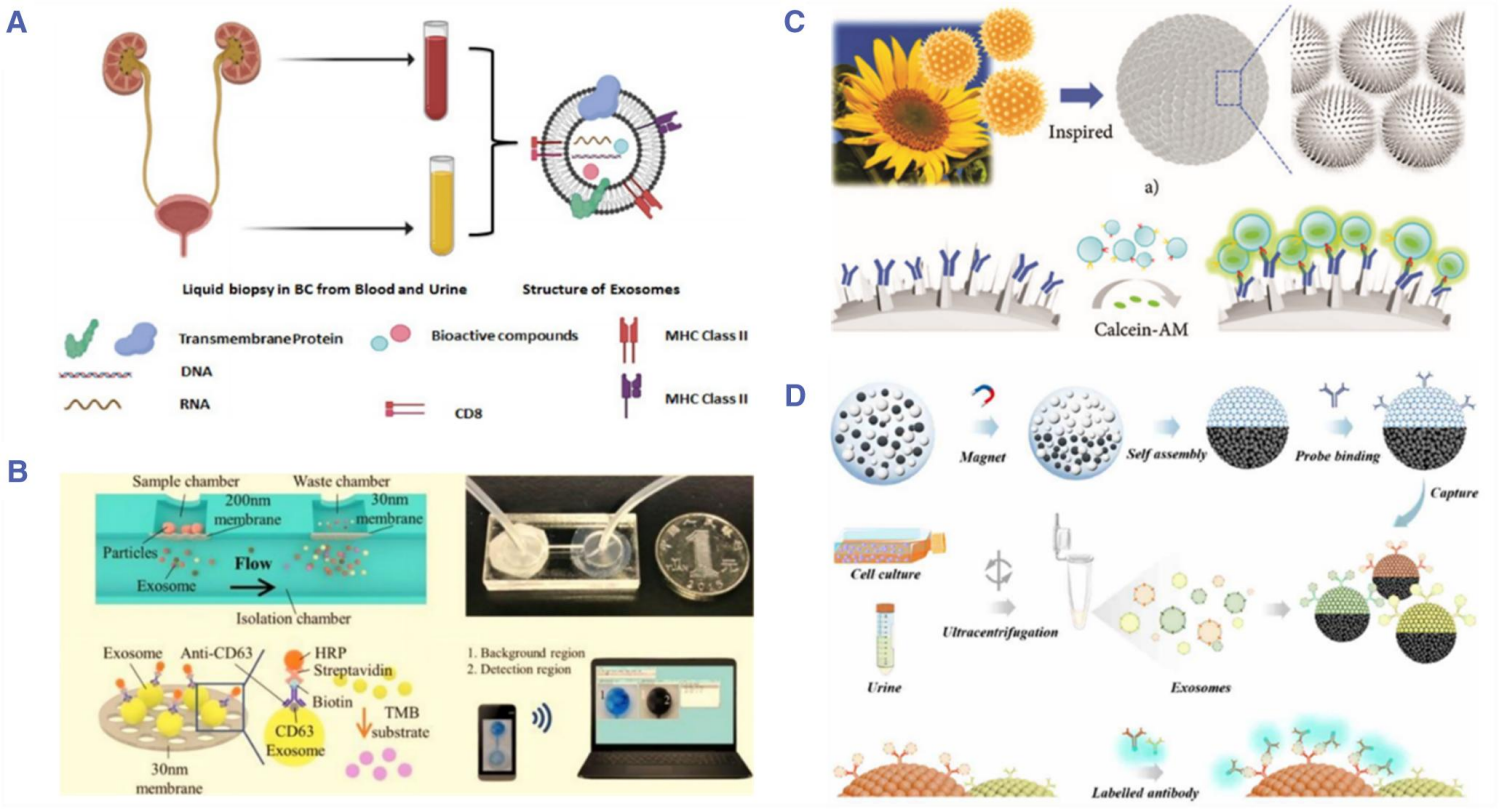
(A) Structure of exosomes as potential source of tumor biomarkers. Reproduced under terms of the CC‐BY license.^111^ Copyright 2021, The Authors, published by MDPI. (B) Separation and identification of EVs from urine using a microfluidic system with integrated double‐filtration. Reproduced under terms of the CC‐BY license.^114^ Copyright 2017, The Authors, published by Springer Nature. (C) Schematic diagram of pollen‐inspired PhC barcode and exosome specific recognition. Reproduced under terms of the CC‐BY license.^115^ Copyright 2022, The Authors, published by AAAS. (D) Schematic diagram of the Janus magnetic microspheres for noninvasive analysis of bladder cancer‐derived urinary exosomes. Reproduced with permission.^116^ Copyright 2022, ACS. EVs, extracellular vesicles.

Exosomes are the tiny endosome derived membrane nanovesicles that play an important role in cell communication.^42^ Currently, nanotechnology has been widely used in tumor‐related research, including in vitro diagnosis, in vivo imaging enhancement and drug loading. As the natural nanoparticles, exosomes exist in most biological fluids. Combined with the advanced biomedical engineering technologies, exosomes show important application potential in the tumor diagnosis and treatment^110^, ^111^, ^112^, ^113^ (Figure 8A). Liang et al. constructed an optimized dual‐filter microfluidic system, which isolated and purified EVs from urine and quantified them by microchip ELISA. Receiver operating characteristic curve analysis of 16 BC patients and 8 healthy controls showed that the integrated system had a sensitivity of 81.3% and a specificity of 90%, and had the expected potential to improve the clinical diagnosis of BC in the combination with urocytology and cystoscopy^114^ (Figure 8B). Inspired by the unique structure of pollen, Li et al. adopted hydrothermal synthesis method to propose a photonic crystal barcode with prickly surface for the capture and screening of multiple exosomes. These pollen‐inspired PhC barcodes had extremely high specific surface area and excellent prickly surface nanostructure, which could improve the exosome capture rate and detection sensitivity^115^ (Figure 8C). In another work, Wei et al. proposed a multiplexed analysis of urinary exosomes from BC based on Janus magnetic microspheres as barcodes. They were composed of colloidal silica nanoparticles and magnetic nanoparticles co‐assembled through a droplet template with both structural color encoding and magnetic responsiveness^116^, ^117^, ^118^ (Figure 8D). These schemes not only effectively enrich BC‐related exosomes, but also support multiple screening of exosomes with high sensitivity, providing new strategies for laboratory diagnosis based on exosomes.

## 4 CONCLUSION

Exosomes, which contain a plethora of active molecular components, serve as crucial mediators for intercellular communication. Their ubiquitous presence in blood and urine renders them efficacious and auspicious biomarkers for clinical diagnosis and prognostic monitoring of various diseases. Furthermore, the bioactive molecules encapsulated within exosomes hold potential as drug carriers and therapeutic targets. Prior researches have indicated that cystoscopy utilizing tissue sampling is the fundamental method for diagnosing BC, while exosome biomarkers possess a heightened sensitivity and can serve as a valuable and encouraging supplementary diagnostic tool in clinical settings. Notably, the isolation of exosomes derived from tumors in patients with low‐grade tumors highlights the potential importance of exosomes as tumor markers in the absence of conventional clinical symptoms, cystoscopy, or imaging results. Furthermore, the utilization of urinary exosome‐derived biomarkers in the clinical detection of BC is advantageous due to the accessibility of urine as a bodily fluid specimen. Although exosome‐based non‐invasive tools and potential therapies have been employed for clinical diagnosis and prognostic monitoring in certain tumors, the current researches on exosomes in BC is still in its preliminary phases. Despite exosomes being a promising candidate for the diagnosis and prognosis of BC, there remain numerous challenges in the areas of mechanism research and technical advancements. Thus, a comprehensive exploration of the biological characteristics and potential clinical applications of BC exosomes is necessary.

## AUTHOR CONTRIBUTIONS

Yefei Zhu and Dagan Zhang conceived the topic of this review; Xiaowei Wei wrote the manuscript and revised the content.

## CONFLICT OF INTEREST STATEMENT

The authors declare that there are no competing interests.

## References

[1] D. M. Jiang , S. Gupta , A. Kitchlu , A. Meraz‐Munoz , S. A. North , N. S. Alimohamed , N. Blais , S. S. Sridhar , Nat. Rev. Urol. 2021, 18, 104. 33432181 10.1038/s41585-020-00404-6

[2] J. H. van Puffelen , S. T. Keating , E. Oosterwijk , A. G. van der Heijden , M. G. Netea , L. A. B. Joosten , S. H. Vermeulen , Nat. Rev. Urol. 2020, 17, 513. 32678343 10.1038/s41585-020-0346-4

[3] C. Berdik , Nature 2017, 551, S34. 29117159 10.1038/551S34a

[4] F. Bray , J. Ferlay , I. Soerjomataram , R. L. Siegel , L. A. Torre , A. Jemal , CA Cancer J. Clin. 2018, 68, 394. 30207593 10.3322/caac.21492

[5] K. C. Degeorge , H. R. Holt , S. C. Hodges , Am. Fam. Physician 2017, 96, 507. 29094888

[6] A. T. Lenis , P. M. Lec , K. Chamie , JAMA 2020, 324, 1980. 33201207 10.1001/jama.2020.17598

[7] A. Saad , D. C. Hanbury , T. A. Mcnicholas , G. B. Boustead , A. C. Woodman , Eur. Urol. 2001, 39, 619. 11464050 10.1159/000052519

[8] J. W. Lee , in Bladder Cancer (Ed: J. H. Ku ), Academic Press, Cambridge, MA 2018, pp. 83–86.

[9] P. Simeone , G. Bologna , P. Lanuti , L. Pierdomenico , M. T. Guagnano , D. Pieragostino , P. Del Boccio , D . Vergara , M. Marchisio , S. Miscia , R. Mariani‐Costantini , Int. J. Mol. Sci. 2020, 21, 2514. 32260425 10.3390/ijms21072514 PMC7178048

[10] T. Huyan , H. Li , H. Peng , J. Chen , R. Yang, W. Zhang, Q. Li , Int. J. Nanomed. 2020, 15, 6485. 10.2147/IJN.S238099 PMC7457829 32922012

[11] G. van Niel , G. D'Angelo , G. Raposo , Nat. Rev. Mol. Cell Biol. 2018, 19, 213. 29339798 10.1038/nrm.2017.125

[12] R. Xu , A. Rai , M. Chen , W. Suwakulsiri , D. W. Greening , R. J. Simpson , Nat. Rev. Clin. Oncol. 2018, 15, 617. 29795272 10.1038/s41571-018-0036-9

[13] L. Jiang , Y. Gu , Y. Du , J. Liu , Mol. Pharmaceutics 2019, 16, 3333. 10.1021/acs.molpharmaceut.9b00409 31241965

[14] R. M. Johnstone , M. Adam , J. R. Hammond , L. Orr , C. Turbide , J. Biol. Chem. 1987, 262, 9412. 3597417

[15] M. Colombo , G. Raposo , C. Théry , Annu. Rev. Cell Dev. Biol. 2014, 30, 255. 25288114 10.1146/annurev-cellbio-101512-122326

[16] W. Mao , K. Wang , Z. Wu , B. Xu , M. Chen , J. Exp. Clin. Cancer Res. 2021, 40, 305. 34583759 10.1186/s13046-021-02114-2 PMC8477471

[17] C. Masaoutis , S. A. Besher , I. Koutroulis , S. Theocharis , Dis. Markers 2020, 2020, 8897833. 32849923 10.1155/2020/8897833 PMC7441435

[18] X. Zhang , X. Yuan , H. Shi , L. Wu , H. Qian , W. Xu , J. Hematol. Oncol. 2015, 8, 83. 26156517 10.1186/s13045-015-0181-x PMC4496882

[19] L. Du , X. Jiang , W. Duan , R. Wang , I. Wang , G. Zheng , K. Yan , L. Wang , J. Li , X. Zhang , H. Pan , Y. Yang , C. Wang , Oncotarget 2017, 8, 40832. 28388561 10.18632/oncotarget.16586 PMC5522322

[20] V. Urquidi , M. Netherton , E. Gomes‐Giacoia , D. J. Serie , J. Eckel‐Passow, C. J. Rosser, S. Goodison , Oncotarget 2016, 7, 86290. 27863434 10.18632/oncotarget.13382 PMC5349914

[21] R. Kalluri , J. Clin. Invest. 2016, 126, 1208. 27035812 10.1172/JCI81135 PMC4811149

[22] L. Han , E. W. Lam , Y. Sun , Mol. Cancer 2019, 18, 59. 30925927 10.1186/s12943-019-0980-8 PMC6441234

[23] Y. Tong , X. Liu , D. Xia , E. Peng , X. Yang , H. Liu , T. Ye , X. Wang , Y. He , H. Xu , Z. Ye , Z. Chen , K. Tang , Front. Oncol. 2021, 11, 704703. 34692482 10.3389/fonc.2021.704703 PMC8530185

[24] C. Kahlert , R. Kalluri , J. Mol. Med. 2013, 91, 431. 23519402 10.1007/s00109-013-1020-6 PMC4073669

[25] D. Choi , T. Lee , C. Spinelli , S. Chennakrishnaiah , E. D'Asti , J. Rak , Semin. Cell Dev. Biol. 2017, 67, 11. 28077296 10.1016/j.semcdb.2017.01.003

[26] C. Grange , M. Tapparo , F. Collino , L. Vitillo , C. Damasco , M. C. Deregibus , C. Tetta , B. Bussolati , G. Camussi , Cancer Res. 2011, 71, 5346. 21670082 10.1158/0008-5472.CAN-11-0241

[27] A. Audia , S. Conroy , R. Glass , K. P. L. Bhat , Front. Oncol. 2017, 7, 143. 28740831 10.3389/fonc.2017.00143 PMC5502267

[28] F. Urabe , N. Kosaka , T. Kimura , S. Egawa , T. Ochiya , Int. J. Urol. 2018, 25, 533. 29726046 10.1111/iju.13594

[29] C. Xue , Y. Shen , X. Li , B. Li , S. Zhao , J. Gu , Y. Chen , B. Ma , J. Wei , Q. Han , R. C. Zhao , Stem Cells Dev. 2018, 27, 456. 29415626 10.1089/scd.2017.0296

[30] C. Grange , A. Brossa , B. Bussolati , Int. J. Mol. Sci. 2019, 20, 1832. 31013896 10.3390/ijms20081832 PMC6514717

[31] K. Jingushi , M. Uemura , N. Ohnishi , W. Nakata , K. Fujita , T. Naito , R. Fujii , N. Saichi , N. Nonomura , K. Tsujikawa , K. Ueda , Int. J. Cancer 2018, 142, 607. 28975613 10.1002/ijc.31080

[32] R. Lugano , M. Ramachandran , A. Dimberg , Cell. Mol. Life Sci. 2020, 77, 1745. 31690961 10.1007/s00018-019-03351-7 PMC7190605

[33] C. A. Franzen , R. H. Blackwell, V. Todorovic, K. A. Greco , K. E. Foreman , R. C. Flanigan, P. C. Kuo, G. N. Gupta , Oncogenesis 2015, 4, e163. 26280654 10.1038/oncsis.2015.21 PMC4632072

[34] T. L. Whiteside , Adv. Clin. Chem. 2016, 74, 103. 27117662 10.1016/bs.acc.2015.12.005 PMC5382933

[35] J. Sceneay , M. J. Smyth , A. Möller , Cancer Metastasis Rev. 2013, 32, 449. 23636348 10.1007/s10555-013-9420-1

[36] Y. Liu , X. Cao , Cancer Cell 2016, 30, 668. 27846389 10.1016/j.ccell.2016.09.011

[37] T. Smyth , M. Kullberg , N. Malik , P. Smith‐Jones , M. W. Graner , T. J. Anchordoquy , J. Controlled Release 2015, 199, 145. 10.1016/j.jconrel.2014.12.013 PMC4441346 25523519

[38] C. F. Ruivo , B. Adem , M. Silva , S. A. Melo , Cancer Res. 2017, 77, 6480. 29162616 10.1158/0008-5472.CAN-17-0994

[39] B. H. Sung , A. M. Weaver , Cell Adhes. Migr. 2017, 11, 187. 10.1080/19336918.2016.1273307 PMC5351719 28129015

[40] V. K. Mishra , M. Subramaniam , V. Kari, K. S. Pitel, S. J. Baumgart , R. M. Naylor, S. Nagarajan, F. Wegwitz, V. Ellenrieder, J. R. Hawse, S. A. Johnsen , Cancer Res. 2017, 77, 2387. 28249899 10.1158/0008-5472.CAN-16-2589 PMC5445903

[41] T. Du , G. Ju , S. Wu , Z. Cheng , J. Cheng , X. Zou , G. Zhang , S. Miao , G. Liu , Y. Zhu , PLoS One 2014, 9, e96836. 24797571 10.1371/journal.pone.0096836 PMC4010513

[42] F. Elsharkawi , M. Elsabah , M. Shabayek , H. Khaled , Asian Pac. J. Cancer Prev. 2019, 20, 2219. 31350988 10.31557/APJCP.2019.20.7.2219 PMC6745236

[43] X. M. Piao , E. J. Cha , S. J. Yun , W. J. Kim , Int. J. Mol. Sci. 2021, 22, 1713. 33567779 10.3390/ijms22041713 PMC7915637

[44] G. Chen , A. C. Huang , W. Zhang , G. Zhang , M. Wu , W. Xu , Z. Yu , J. Yang , B. Wang , H. Sun , H. Xia , Q. Man , W. Zhong , L. F. Antelo , B. Wu , X. Xiong , X. Liu , L. Guan , T. Li , S. Liu , R. Yang , Y. Lu , L. Dong , S. McGettigan , R. Somasundaram , R. Radhakrishnan , G. Mills , Y. Lu , J. Kim , Y. H. Chen , H. Dong , Y. Zhao , G. C. Karakousis , T. C. Mitchell , L. M. Schuchter , M. Herlyh , E. J. Wherry , X. Xu , W. Guo , Nature 2018, 560, 382. 30089911 10.1038/s41586-018-0392-8 PMC6095740

[45] M. Poggio , T. Hu , C. C. Pai , B. Chu , C. D. Belair , A. Chang , E. Montabana , U. E. Lang , Q. Fu , L. Fong , R. Blelloch , Cell 2019, 177, 414. 30951669 10.1016/j.cell.2019.02.016 PMC6499401

[46] C. A. Franzen , R. H. Blackwell , K. E. Foreman , P. C. Kuo , R. C. Flanigan , G. N. Gupta , J. Urol. 2016, 195, 1331. 26714199 10.1016/j.juro.2015.08.115

[47] C. Xiao , K. Rajewsky , Cell 2009, 136, 26. 19135886 10.1016/j.cell.2008.12.027

[48] A. Taghikhani , Z. M. Hassan , M. Ebrahimi , S. M. Moazzeni , J. Cell. Physiol. 2018, 234, 9417. 30362582 10.1002/jcp.27626

[49] K. B. Johnsen , J. M. Gudbergsson , M. N. Skov , L. Pilgaard , T. Moos , M. Duroux , Biochim. Biophys. Acta 2014, 1846, 75. 24747178 10.1016/j.bbcan.2014.04.005

[50] P. Yingchoncharoen , D. S. Kalinowski , D. R. Richardson , Pharmacol. Rev. 2016, 68, 701. 27363439 10.1124/pr.115.012070 PMC4931871

[51] H. Zhou , T. Pisitkun , A. Aponte , P. S. T. Yuen , J. D. Hoffert , H. Yasuda , X. Hu , L. Chawla , R. F. Shen , M. A. Knepper , R. A. Star , Kidney Int. 2006, 70, 1847. 17021608 10.1038/sj.ki.5001874 PMC2277342

[52] A. Pérez , A. Loizaga , R. Arceo , I. Lacasa , A. Rabade , K. Zorroza , D. Mosen‐Ansorena , E. Gonzalez , A. M. Aransay , J. M. Falcon‐Perez , M. Unda‐Urzaiz , F. Royo , Cancers 2014, 6, 179. 24458310 10.3390/cancers6010179 PMC3980604

[53] F. Wahid , A. Shehzad , T. Khan , Y. Y. Kim , Biochim. Biophys. Acta 2010, 1803, 1231. 20619301 10.1016/j.bbamcr.2010.06.013

[54] M. C. de Oliveira , H. R. Caires , M. J. Oliveira , A. Fraga , M. H. Vasconcelos, R. Ribeiro , Cancers 2020, 12, 1400. 32485907 10.3390/cancers12061400 PMC7352974

[55] S. Zhang , L. Du , L. Wang , X. Jiang , Y. Zhan , J. Li , K. Yan , W. Duan , Y. Zhao , L. Wang , Y. Wang , Y. Shi , C. Wang , J. Cell Mol. Med. 2019, 23, 1396. 30467945 10.1111/jcmm.14042 PMC6349164

[56] A. Mari , R. Campi , R. Tellini , G. Gandaglia , S. Albisinni , M. Abufaraj , G. Hatzichristodoulou , F. Montorsi , R. Velthoven , M. Carini , A. Minervini , S. F. Shariat , World J. Urol. 2018, 36, 157. 29147759 10.1007/s00345-017-2115-4 PMC5799348

[57] K. Matsuzaki , K. Fujita , K. Jingushi , A. Kawashima , T. Ujike, A. Nagahara, Y. Ueda, G. Tanigawa, I. Yoshioka, K. Ueda, R. Hanayama, M. Uemura, Y. Miyagawa, K. Tsujikawa, N. Nonomura , Oncotarget 2017, 8, 24668. 28160564 10.18632/oncotarget.14969 PMC5421878

[58] L. Barile , G. Vassalli , Pharmacol. Ther. 2017, 174, 63. 28202367 10.1016/j.pharmthera.2017.02.020

[59] Q. Li , T. Huyan , S. Cai , Q. Huang , M. Zhang , H. Peng , Y. Zhang , N. Liu , W. Zhang , FASEB J. 2020, 34, 12177. 32716585 10.1096/fj.202000347R

[60] X. D. Xu , X. H. Wu , Y. R. Fan , B. Tan , Z. Quan , C. L. Luo , Asian Pac. J. Cancer Prev. 2014, 15, 3471. 24870742 10.7314/apjcp.2014.15.8.3471

[61] E. A. Gibb , C. J. Brown , W. L. Lam , Mol. Cancer 2011, 10, 38. 21489289 10.1186/1476-4598-10-38 PMC3098824

[62] S. Baumgart , P. Meschkat , P. Edelmann , J. Heinzelmann , A. Pryalukhin , R. Bohle , J. Heinzelbecker , M. Stöckle , K. Junker , *J. Cancer Res.* Clin. Oncol. 2019, 145, 2725. 10.1007/s00432-019-03035-6 PMC11810427 31552489

[63] C. Berrondo , J. Flax , V. Kucherov , A. Siebert , T. Osinski , A. Rosenberg , C. Fucile , S. Richheimer , C. J. Beckham , PLoS One 2016, 11, e0147236. 26800519 10.1371/journal.pone.0147236 PMC4723257

[64] Y. Fang , M. J. Fullwood , Genomics Proteomics Bioinf. 2016, 14, 42. 10.1016/j.gpb.2015.09.006 PMC4792843 26883671

[65] A. G. van der Heijden , J. A. Witjes , Eur. Urol. Suppl. 2009, 8, 556.

[66] M. Xue , W. Chen , A. Xiang , R. Wang , H. Chen , J. Pan , H. Pang , H. An , X. Wang , H. Hou , X. Li , Mol. Cancer 2017, 16, 143. 28841829 10.1186/s12943-017-0714-8 PMC5574139

[67] C. Chen , Y. Luo , W. He , Y. Zhao , Y. Kong , H. Liu , G. Zhong , Y. Li , J. Li , J. Huang , R. Chen , T. Lin , J. Clin. Invest. 2020, 130, 404. 31593555 10.1172/JCI130892 PMC6934220

[68] H. Zheng , C. Chen , Y. Luo , M. Yu , W. He , M. An , B. Gao , Y. Kong , Y. Ya , Y. Lin , Y. Li , K. Xie , J. Hang , T. Lin , Clin. Transl. Med. 2021, 11, e497. 34323412 10.1002/ctm2.497 PMC8288020

[69] C. S. Huang , J. Y. Ho , J. H. Chiang , C. P. Yu , D. S. Yu , Cells 2020, 9, 1419.32517366

[70] F. Yazarlou , S. J. Mowla , V. K. Oskooei , E. Motevaseli , L. F. Tooli , M. Afsharpad , L. Nekoohesh , N. S. Sanikhani , S. Ghafouri‐Fard , M. H. Modarressi , Cancer Manag. Res. 2018, 10, 5373. 30464633 10.2147/CMAR.S180389 PMC6225912

[71] S. Mathivanan , R. J. Simpson , Proteomics 2009, 9, 4997. 19810033 10.1002/pmic.200900351

[72] Y. Xia , B. Li , N. Gao , H. Xia , Y. Men , Y. Liu , Z. Liu , Q. Chen , L. Li , Oncol. Lett. 2014, 8, 1670. 25202389 10.3892/ol.2014.2400 PMC4156178

[73] C. L. Chen , Y. F. Lai , P. Tang , K. Y. Chien , J. S. Yu , C. H. Tsai , H. W. Chen , C. C. Wu , T. Chung , C. W. Hsu , C. D. Chen , Y. S. Chang , P. L. Chang , Y. T. Chen , J. Proteome Res. 2012, 11, 5611. 23082778 10.1021/pr3008732

[74] Y. Zhan , Y. Li , B. Guan , Z. Wang , D. Peng , Z. Chen , A. He , S. He , Y. Gong , X. Li , L. Zhou , Oncotarget 2017, 8, 76656. 29100339 10.18632/oncotarget.20795 PMC5652733

[75] A. Li , T. Zhang , M. Zheng , Y. Liu , Z. Chen , J. Hematol. Oncol. 2017, 10, 175. 29282096 10.1186/s13045-017-0542-8 PMC5745959

[76] J. L. Welton , S. Khanna , P. J. Giles , P. Brennan , I. A. Brewis , J. Staffurth , M. D. Mason , A. Clayton , Mol. Cell. Proteomics 2010, 9, 1324. 20224111 10.1074/mcp.M000063-MCP201 PMC2877990

[77] A. Shvartsur , B. Bonavida , Genes Cancer 2015, 6, 84. 26000093 10.18632/genesandcancer.40 PMC4426947

[78] E. Hosseini‐Beheshti , S. Pham , H. Adomat , N. Li , E. S. T. Guns , Mol. Cell. Proteomics 2012, 11, 863. 22723089 10.1074/mcp.M111.014845 PMC3494141

[79] L. M. Doyle , M. Z. Wang , Cells 2019, 8, 727.31311206

[80] N. Georgantzoglou , A. Pergaris , C. Masaoutis , S. Theocharis , Int. J. Mol. Sci. 2021, 22, 2744. 33803085 10.3390/ijms22052744 PMC7963171

[81] L. Yang , X. Wu , D. Wang , C. Luo , L. Chen , Mol. Med. Rep. 2013, 8, 1272. 23969721 10.3892/mmr.2013.1634

[82] M. S. Ostenfeld , D. K. Jeppesen , J. R. Laurberg , A. T. Boysen , J. B. Bramsen , B. Primdal‐Bengtson , A. Hendrix , P. Lamy , F. Dagnaes‐Hansen , M. H. Rasmussen , K. H. Bui , N. Fristrup , E. I. Christensen , I. Nordentoft , J. P. Morth , J. B. Jensen , J. S. Pedersen , M. Beck , D. Theodorescu , M. Borre , K. A. Howard , L. Dyrskøt , T. F. Ørntoft , Cancer Res. 2014, 74, 5758. 25261234 10.1158/0008-5472.CAN-13-3512

[83] C. J. Beckham , J. Olsen , P. N. Yin , C. H. Wu , H. J. Ting , F. K. Hagen , E. Scosyrev , E. M. Messing , Y. F. Lee , J. Urol. 2014, 192, 583. 24530986 10.1016/j.juro.2014.02.035

[84] A. Di Meo , J. Bartlett , Y. Cheng , M. D. Pasic , G. M. Yousef , Mol. Cancer 2017, 16, 80. 28410618 10.1186/s12943-017-0644-5 PMC5391592

[85] S. Baumgart , S. Hölters , C. H. Ohlmann , R. Bohle , M. Stöckle , M. S. Ostenfeld , L. Dyrskjøt , K. Junker , J. Heinzelmann , Oncotarget 2017, 8, 58278. 28938555 10.18632/oncotarget.17619 PMC5601651

[86] Z. Andreu , R. O. Oshiro , A. Redruello , S. López‐Martín , C. Gutiérrez‐Vázquez , E. Morato , A. I. Marina , C. O. Gómez , M. Yáñez‐Mó , Eur. J. Pharm. Sci. 2017, 98, 70. 27751843 10.1016/j.ejps.2016.10.008

[87] S. M. Kim , H. W. Kang , W. T. Kim , Y. J. Kim , S. J. Yun , S. C. Lee , W. J. Kim , Korean J. Urol. 2013, 54, 791. 24255763 10.4111/kju.2013.54.11.791 PMC3830974

[88] N. Sapre , G. Macintyre , M. Clarkson , H. Naeem , M. Cmero , A. Kowalczyk , P. D. Anderson , A. J. Costello , N. M. Corcoran , C. M. Hovens , Br. J. Cancer 2016, 114, 454. 26812572 10.1038/bjc.2015.472 PMC4815774

[89] X. Jiang , L. Du , L. Wang , J. Li , Y. Liu , G. Zheng , A. Qu , X. Zhang , H. Pan , Y. Yang , C. Wang , Int. J. Cancer 2015, 136, 854. 24961907 10.1002/ijc.29041

[90] X. Jiang , L. Du , W. Duan , R. Wang , K. Yan , L. Wang, J. Li, G. Zheng, X. Zhang, Y. Yang, C. Wang, Oncotarget 2016, 7, 36733. 27167342 10.18632/oncotarget.9166 PMC5095035

[91] H. Fanous , T. Sullivan , K. Rieger‐Christ , J. Urol. 2017, 197, e1179.

[92] X. Yu , R. Wang , C. Han , Z. Wang , X. Jin , J. Cancer 2020, 11, 781. 31949480 10.7150/jca.37006 PMC6959012

[93] D. A. Armstrong , B. B. Green , J. D. Seigne , A. R. Schned , C. J. Marsit , Mol. Cancer 2015, 14, 194. 26576778 10.1186/s12943-015-0466-2 PMC4650939

[94] Y. Zhan , L. Du , L. Wang , X. Jiang , S. Zhang , J. Li , K. Yan , W. Duan , Y. Zhao , L. Wang , Y. Wang , C. Wang , Mol. Cancer 2018, 17, 142. 30268126 10.1186/s12943-018-0893-y PMC6162963

[95] R. Zheng , M. Du , X. Wang , W. Xu , J. Liang, W. Wang, Q. Lv, C. Qin, H. Chu, M. Wang, L. Yuan, J. Qian, Z. Zhang , Mol. Cancer 2018, 17, 143. 30285771 10.1186/s12943-018-0880-3 PMC6169076

[96] J. Wang , K. Yang , W. Yuan , Z. Gao , Med. Sci. Monit. 2018, 24, 9307. 30576305 10.12659/MSM.912018 PMC6320644

[97] C. R. Silvers , Y. R. Liu , C. H. Wu , H. Miyamoto , E. M. Messing, Y. F. Lee , Oncotarget 2016, 7, 23335. 26981774 10.18632/oncotarget.8024 PMC5029630

[98] S. Y. Lin , C. H. Chang , H. C. Wu , C. C. Lin , K. P. Chang , C. R. Yang , C. P. Huang , W. H. Hsu , C. T. Chang , C. J. Chen , Sci. Rep. 2016, 6, 34446. 27686150 10.1038/srep34446 PMC5043375

[99] M. C. Henderson , D. O. Azorsa , Front. Oncol. 2012, 2, 38. 22649786 10.3389/fonc.2012.00038 PMC3355967

[100] K. Liang , F. Liu , J. Fan , D. Sun , C. Liu , C. J. Lyon , D. W. Bernard , Y. Li , K. Yokoi , M. H. Katz , E. J. Koay , Z. Zhao , Y. Hu , Nat. Biomed. Eng. 2017, 1, 0021. 10.1038/s41551-016-0021 PMC5543996 28791195

[101] D. K. Jeppesen , A. Nawrocki , S. G. Jensen , K. Thorsen , B. Whitehead , K. A. Howard , L. Dyrskjøt , T. F. Ørntoft , M. R. Larsen , M. S. Ostenfeld , Proteomics 2014, 14, 699. 24376083 10.1002/pmic.201300452

[102] C. R. Silvers , H. Miyamoto , E. M. Messing , G. J. Netto , Y. F. Lee , Oncotarget 2017, 8, 91199. 29207636 10.18632/oncotarget.20043 PMC5710916

[103] Y. Z. Almallah , C. D. Rennie , J. Stone , M. J. R. Lancashire , Urology 2000, 56, 37. 10869618 10.1016/s0090-4295(00)00555-0

[104] D. K. Jeppesen , A. M. Fenix , J. L. Franklin , J. N. Higginbotham , Q. Zhang , L. J. Zimmerman , D. C. Liebler , J. Ping , Q. Liu , R. Evans , W. H. Fissell , J. G. Patton, L. H. Rome , D. T. Burnette , R. J. Coffey , Cell 2019, 177, 428. 30951670 10.1016/j.cell.2019.02.029 PMC6664447

[105] Y. Wang , J. Liu , J. Ma , T. Sun , Q. Zhou , W. Wang , G. Wang , P. Wu , H. Wang , L. Jiang , W. Yuan , Z. Sun , L. Ming , Mol. Cancer 2019, 18, 116. 31277663 10.1186/s12943-019-1041-z PMC6610963

[106] T. A. Shtam , R. A. Kovalev , E. Y. Varfolomeeva , E. M. Makarov , Y. V. Kil , M. V. Filatov , Cell Commun. Signal. 2013, 11, 88. 24245560 10.1186/1478-811X-11-88 PMC3895799

[107] Z. Wu , Z. Zhang , W. Xia , J. Cai , Y. Li , S. Wu , Cell Proliferation 2019, 52, e12659. 31469460 10.1111/cpr.12659 PMC6869217

[108] C. A. Franzen , P. E. Simms , A. F. Van Huis , K. E. Foreman , P. C. Kuo , G. N. Gupta , BioMed Res. Int. 2014, 2014, 619829. 24575409 10.1155/2014/619829 PMC3915764

[109] K. A. Greco , C. A. Franzen , K. E. Foreman , R. C. Flanigan , P. C. Kuo , G. N. Gupta , Urology 2016, 91, 241.e1. 10.1016/j.urology.2016.01.028 26876462

[110] R. Szatanek , M. Baj‐Krzyworzeka , J. Zimoch , M. Lekka , M. Siedlar , J. Baran , Int. J. Mol. Sci. 2017, 18, 1153. 28555055 10.3390/ijms18061153 PMC5485977

[111] M. Barani , S. M. Hosseinikhah , A. Rahdar , L. Farhoudi , R. Arshad , M. Cucchiarini , S. Pandey , Cancers 2021, 13, 2214. 34063088 10.3390/cancers13092214 PMC8125468

[112] J. G. Che , A. Najer , A. K. Blakney , P. F. McKay , M. Bellahcene , C. W. Winter , A. Sintou , J. Tang , T. J. Keane , M. D. Schneider , R. J. Shattock , S. Sattler , M. M. Stevens , Adv. Mater. 2020, 32, 2003598. 10.1002/adma.202003598 PMC7613371 33103807

[113] J. D. Friedl , V. Nele , G. De Rosa , A. Bernkop‐Schnürch , Adv. Funct. Mater. 2021, 31, 2103347.

[114] L. G. Liang , M. Q. Kong , S. Zhou , Y. F. Sheng , P. Wang , T. Yu , F. Inci , W. P. Kuo , L. J. Li , U. Demirci , S. Wang , Sci. Rep. 2017, 7, 46224. 28436447 10.1038/srep46224 PMC5402302

[115] N. Li , F. Bian , X. Wei , L. Cai , H. Gu , Y. Zhao , L. Shang , Research 2022, 2022, 9809538. 36128177 10.34133/2022/9809538 PMC9470204

[116] X. Wei , L. Cai , H. Chen , L. Shang , Y. Zhao , W. Sun , Anal. Chem. 2022, 94, 18034. 36519619 10.1021/acs.analchem.2c04408

[117] B. Ye , F. Rong , H. Gu , Z. Xie , Y. Cheng , Y. Zhao , Z. Gu , Chem. Commun. 2013, 49, 5331. 10.1039/c3cc42122h 23649037

[118] L. Shang , F. Shangguan , Y. Cheng , J. Lu , Z. Xie , Y. Zhao , Z. Gu , Nanoscale 2013, 5, 9553. 23979459 10.1039/c3nr03218c

